# The performance of an atomically dispersed oxygen reduction catalyst prepared by γ-CD-MOF integration with FePc†

**DOI:** 10.1039/d2na00095d

**Published:** 2022-04-11

**Authors:** Dawei Xu, Xuhui Li, Tingting Zheng, Ruixue Zhao, Pengyu Zhang, Kai Li, Zhongfeng Li, Lirong Zheng, Xia Zuo

**Affiliations:** Department of Chemistry, Capital Normal University Beijing 100048 China zuoxia@cnu.edu.cn +86-10-68903040 +86-10-68903086; Department Beijing Synchrotron Radiation, Facility Institute of High Energy Physics China

## Abstract

Here, a series of Fe/N/C catalysts with different proportions and pyrolysis temperatures are prepared by co-pyrolysis of melamine with a γ-cyclodextrin metal–organic framework (γ-CD-MOF) containing iron(ii) phthalocyanine (FePc). Due to the restriction effect of the host and guest at the molecular level, γ-CD-MOF can effectively avoid the π–π stacking of FePc and restrain the agglomeration of Fe atoms during pyrolysis. The phases and structures of the catalysts are characterized, which proves that the obtained catalyst has a three-dimensional porous and internal cavity structure with abundant surface area (1055.317 m^2^ g^−1^) and Fe is atomically dispersed in nitrogen-doped carbon. The onset potential (0.988 V *vs.* RHE) and half-wave potential (0.846 V *vs.* RHE) of FePc@CD/M (1 : 20)-1000 are superior to those of a commercial 20% Pt/C catalyst. FePc@CD/M (1 : 20)-1000 also exhibits an approximately four-electron (3.84) transfer process, good stability and excellent methanol tolerance.

## Abstract 2

The limiting effect of the γ-CD-MOF cavity can prevent the accumulation of FePc and inhibit the agglomeration of Fe, thus yielding an atomically dispersed Fe/N/C electrocatalyst.

## Graphical abstract

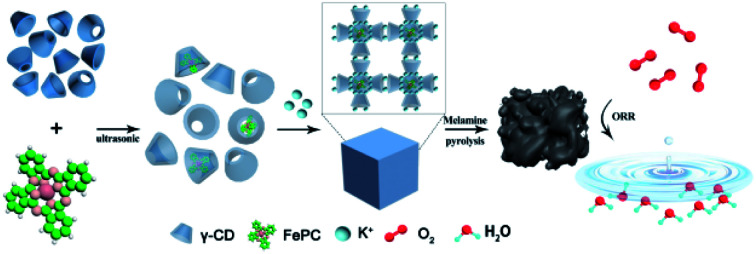

## 1. Introduction

Fuel cells (FCs) as clean and sustainable energy conversion devices are one of the important solutions to the current environmental problems. Improving the slow oxygen reduction reaction (ORR) kinetics is the key to achieving high efficiency and power density in fuel cells.^1,2^ At present, Pt-based materials are the most applicable cathode catalysts, but its low reserves and high cost hinder the wide application of fuel cells.^3–5^ Therefore, it is urgent to explore efficient non-noble metal catalyst alternatives. Non-precious metal-doped nanocatalysts, such as graphene–Co_3_O_4_ nanocomposites,^6^ have shown comparable ORR activity to Pt, and non-precious metal ORR catalysts show better durability under relatively mild conditions. Most catalysts based on non-noble metals exhibit good catalytic performance in alkaline electrolyte and high methanol tolerance.^7–9^ For non-noble metal catalysts, it is necessary not only to improve the catalytic performance, but also to overcome the problems of complex preparation process, high temperature calcination, and unstable batch performance in order to achieve application. In theory, the active centers of non-noble metal catalysts are not clear,^10,11^ and more advanced *in situ* test instruments and technologies need to be explored. Many current studies of transition metal monoatomic catalysts, such as Fe–N–C doped mesoporous carbon frameworks,^12^ have used a combination of experimental and theoretical calculations to identify the active site of the ORR. In the long run, reducing costs and developing non-platinum catalysts with high activity and durability will be an important direction for future research on cathode oxygen reduction catalysts.

Since Raymond Jasinski first proposed and reported the activity of CoPc as a cathode catalyst for fuel cells in 1964,^13^ the use of transition metal–N_4_ (M–N_4_) macrocyclic complexes in catalytic ORR has attracted wide attention. Well-defined M–N_4_ macrocyclic molecules, such as metal phthalocyanines (MPcs)^14,15^ and metalloporphyrins (MPs),^16,17^ have a specific M–N_4_ coordination structure, which provides an excellent model catalyst for studying the active center and reaction mechanism. However, due to the π–π conjugation effect, phthalocyanines easily aggregate.^18^ After pyrolysis, the central metal easily separates and agglomerates, and its use is often faced with serious problems, resulting in low activity and durability.

Porous materials represented by covalent organic frameworks and metal organic frameworks have become the focus of many researchers due to their extensive applications in gas storage,^19^ molecular separation^20^ and catalysis.^21^ With their unique structural properties, MOFs can effectively help metal atoms achieve good dispersion in the preparation of catalytic materials. Wei *et al.* prepared rho-ZIF that was used to encapsulate Fe-TPP molecules by ball milling, and then pyrolyzed it to form an atomically dispersed Fe/N/C catalyst.^22^ However, the selectivity of sod- and rho-ZIF in the synthesis process will lead to the loss of target precursor yield. Moreover, the residual Fe-TPP was attached on the surface of rho-ZIF, which increased the impurity removal process. Porous organic cage γ-CD-MOF is formed by cross-linking of hydroxyl and potassium ions in a cyclodextrin skeleton. With both the cage structure and cavity structure, CD-MOFs can provide an extremely rich host environment to load a variety of guest molecules. Gong *et al.* used organic dyes rhodamine B (RHB) and methylene blue (MB) as guest components and prepared γ-CD-MOF by a standard solvent diffusion method, where the object is encapsulated in a (γ-CD)_6_ cube for effective adsorption and release.^23^ Thus, we are inspired to apply it to the separation of macrocyclic molecules.

Based on the adaptation of phthalocyanine (14.6 1 Å)^24^ and the internal cavity of γ-CD-MOF (16.9 Å),^23^ a strategy of dispersing and anchoring iron phthalocyanine by cyclodextrin (γ-CD) was designed. An Fe/N co-doped porous carbon nanocatalyst was prepared by co-pyrolysis of FePc-coated γ-CD-MOFs with melamine. The phthalocyanine molecules were encapsulated by the hydrophobic cavity structure of cyclodextrin molecules. Due to the restriction effect of the host and guest at the molecular level, the Fe center embedded in γ-CD-MOF could be effectively dispersed during the pyrolysis process to prepare the atomically dispersed Fe–N part anchored on the carbon matrix.

## 2. Experimental section

### 2.1 Chemicals and materials

Chemicals were purchased from commercial sources and used without further purification. The organic solvents used were of analytical grade. γ-Cyclodextrin (Innochem), KOH (Acros), tetrahydrofuran (Innochem), absolute ethyl alcohol (Beijing Chemical Reagent Factory) and iron(ii) phthalocyanine (Aladdin, Shanghai, China) were purchased. Nafion (5%) and a commercial 20 wt% Pt/C electrocatalyst were supplied by Sigma-Aldrich.

### 2.2 Preparation of catalysts

#### 2.2.1 Preparation of FePc@γ-CD-MOF

First, 0.3 mmol of FePc was dissolved in 20.0 mL of 98% H_2_SO_4_ with sonication for 5 min. Then, the violet black solution was added to 80 mL of THF dropwise with sonication in an ice-water bath. In addition, 0.6 mmol of the γ-CD was dissolved in 30.0 mL of 3 M H_2_SO_4_. The two solutions were slowly mixed at room temperature and sonicated in an ice-water bath for 3 h. After sonication, ethanol was added to the solution and a solid precipitated due to the addition of ethanol in a process similar to recrystallisation. Then, the mixture was centrifuged and the precipitate was washed 3 times with ethanol and dried at 60 °C in a constant temperature oven. After that, one equivalent of the dried product was dissolved in eight equivalents of KOH solution and stirred magnetically at room temperature for 24 hours. Then, ethanol was slowly added to the solution to precipitate the solid. Then, the mixture was centrifuged and the precipitate was washed with ethanol. The precipitate was dried at 60 °C overnight to yield the precursor, named FePc@γ-CD-MOF.

#### 2.2.2 Preparation of the Fe/N/C electrocatalyst

The as-prepared precursor powder and melamine were uniformly doped at different equivalent ratios by a conventional grinding method to obtain FePc@γ-CD-MOF/melamine (1 : m). Afterwards, the resultant powder was annealed at 800 °C, 900 °C, 1000 °C, and 1100 °C for 2 h under an argon atmosphere at a ramp rate of 3 °C min^−1^. Eventually the collected powder was stirred in 0.5 M H_2_SO_4_ solution for 12 h, filtered and washed with deionized water until the filtrate was neutral. The obtained powder was dried at 60 °C overnight. The final product is named FePc@CD/M (1 : m)-*T* (*T* = 800, 900, 1000, or 1100 °C).

### 2.3 Materials characterization

SEM images were recorded and EDS mapping analyses performed on Hitachi equipment (Japan). The morphology of the samples was characterized by transmission electron microscopy (TEM, Hitachi, Japan). High-resolution TEM (HRTEM) and elemental mapping images were obtained from a scanning transmission electron microscope (STEM) unit with a high-angle annular dark field (HAADF) detector (FEI, Tecnai, F30, USA). TEM samples were dispersed in ethanol and added dropwise onto carbon supported films. X-ray diffraction (XRD) patterns were recorded on a D8 Advance/Bruker diffractometer (Germany) employing a Cu Kα radiation source (*λ* = 0.1541 nm). X-ray photoelectron spectroscopy (XPS) (ESCALAB 250, UK) was performed using monochromatic Al Kα radiation as the excitation source. X-ray absorption near edge structure (XANES) and extended X-ray absorption fine structure (EXAFS) constituted XAFS spectroscopy and were performed at the Beijing Synchrotron Radiation Facility. The absorption spectra of Fe K-edge spectra were collected in the fluorescence mode using a double-crystal Si(111) monochromator. The pore properties of the samples were determined by using N_2_ adsorption–desorption isotherms recorded on a NOVA 1000e. The Brunauer–Emmett–Teller (BET) specific surface areas were calculated from the isotherms using the BET equation. Raman spectra of all carbonization products were recorded on a confocal Raman microscope (InVia Reflex, Renishaw) equipped with a 633 nm laser emitting source.

### 2.4 Electrochemical measurements

All electrochemical measurements were performed using a three-electrode system controlled by an SP-200 electrochemical station (BioLogic SA, France). The reference electrode was a saturated calomel electrode (SCE) and the counter electrode was a platinum sheet electrode. The working electrode was a rotating ring-disk electrode (RRDE) (Pine Research Instrumentation, USA). The disk electrode was a glass carbon disk with a diameter of 5.0 mm and the ring electrode was a platinum ring with an inner diameter of 6.5 mm and an outer diameter of 7.5 mm. The areas of the disk electrode and the ring electrode were 0.196 and 0.110 cm^−2^, respectively.

For the electrochemical test in 0.1 M KOH, 3 mg of the catalysts was dispersed by sonication for 1 h in 0.5 mL 5 wt% Nafion/ethanol (10 μL + 490 μL). Then 10 μL catalyst ink was loaded on a glassy carbon electrode and dried. Cyclic voltammetry (CV) curves were recorded in an Ar or O_2_-saturated electrolyte, LSV polarization curves were obtained in an O_2_-saturated electrolyte at rotation speeds of 400, 625, 900, 1225, and 1600 rpm, and chronoamperometric curves were recorded in O_2_-saturated solution. The electrochemical impedance spectrum (EIS) was measured from 100 Hz to 100 mHz at low to high frequencies with an applied voltage of 0.83 V and a modulated signal amplitude of 5 mV. To obtain the specific capacitance, the CV curves of the Faraday-free process were recorded at scan rates of 5, 10, 15, 20, 30, 40 and 50 mV s^−1^. The specific capacitance was obtained from the linear slope of the current density *versus* scan rate. In addition, for evaluating the methanol tolerance of the catalysts, methanol (3 M) was added to the electrolyte during the chronoamperometric determination. In this work, all the measured potentials (*vs.* SCE) were converted to the RHE scale:^25,26^*E*_RHE_ = *E*_SCE_ + 0.0591 × pH + 0.244
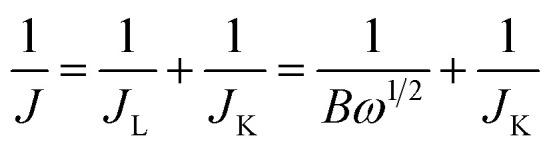
*B* = 0.62*nFC*_0_*D*_0_^2/3^*ν*^−1/6^where *J* is the measured current density, *J*_K_ is the kinetic current density, *J*_L_ is the diffusion-limiting current density, *ω* is the electrode rotation rate (rad s^−1^), *n* is the electron transfer number in the ORR, *F* is the Faraday constant (*F* = 96 485 C mol^−1^), *D*_0_ is the diffusion coefficient of O_2_ (1.9 × 10^−5^ cm^2^ s^−1^ for 0.1 M KOH solution), *C*_0_ is the bulk concentration of O_2_ (1.2 × 10^−6^ mol cm^−3^ for 0.1 M KOH solution) and *ν* is the kinematic viscosity of the electrolyte (*ν* = 1.07 × 10^−2^ cm^2^ s^−1^).

For the RRDE measurements, the disk electrode was also scanned with a rate of 5 mV s^−1^ at a constant ring potential of 1.4 V *vs.* RHE. The peroxide percentage (H_2_O_2_ yields) and the number of electrons transferred (*n*) were calculated according to the following equations:^27,28^
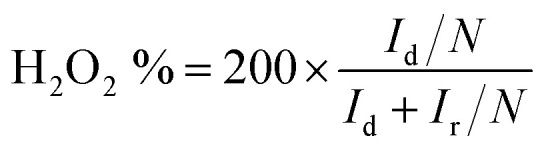

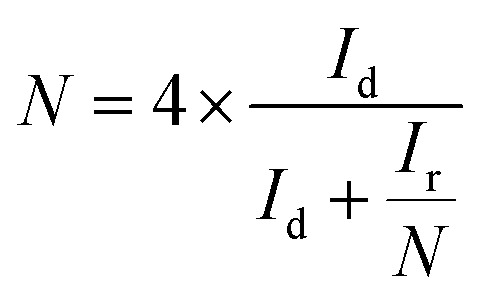
where *I*_d_ is the disk current, and *I*_r_ refers to the ring current and *N* represents the current collection efficiency of the Pt ring (*N* = 0.37).

## 3. Results and discussion

**Scheme 1:**
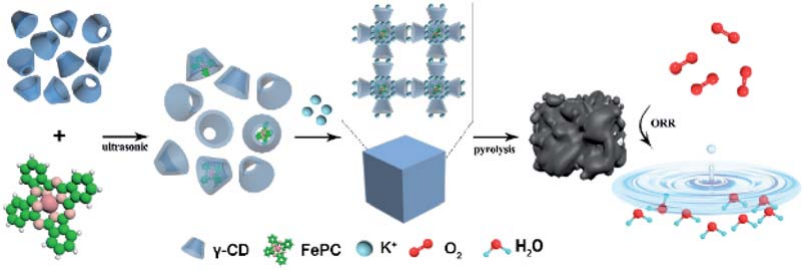
Synthesis method of encapsulating iron phthalocyanine molecules with γ-CD-MOF to prepare the catalysts.

Scheme 1 illustrates the synthesis of Fe/N/C catalysts. FePc molecules are encapsulated in the internal cavity of γ-CD-MOF molecules. Due to the restriction effect of the host and guest at the molecular level, not only is the π–π stacking of FePc inhibited, but the agglomeration of Fe atoms in the pyrolysis process is also avoided, thus yielding atomically dispersed Fe on the carbon carrier.

**Fig. 1:**
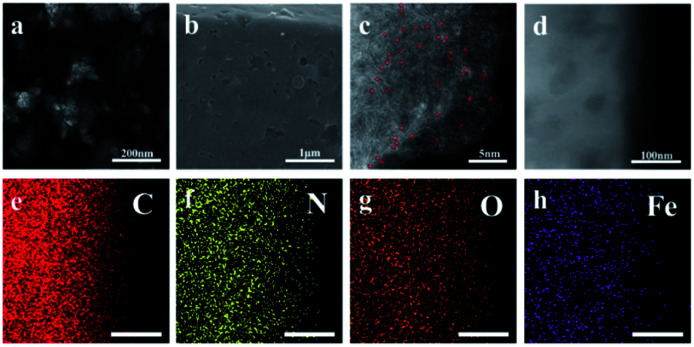
(a) TEM image, (b) SEM image, (c) HAADF-STEM image (the Fe atoms are highlighted by red circles), (d) HRTEM image, and (e–h) elemental mapping images of FePc@CD/M (1 : 20)-1000.

The alternation of light and dark regions in the TEM image (Fig. 1a and S1†) confirmed that the obtained FePc@CD/M (1 : 20)-1000 had an interconnected cavity structure. No obvious metal aggregation was observed in the image, indicating that no Fe nanoparticles and metal oxide particles were formed on the carbon matrix. The SEM image (Fig. 1b) showed that there were obvious pores with different sizes on the surface of the carbon sheets with irregular shapes, which further confirmed its porous characteristics. These irregular carbon sheets are mainly formed by annealing of γ-CD-MOF, and have hollow cavities with exposed inner surfaces. In addition, we also studied the effects of different annealing temperatures and different doping ratios on the internal and surface morphologies of the catalysts (Fig. S2 and S3†). Fig. S2† shows the TEM images of different catalysts, and no Fe agglomerates or metal oxide particles were formed in the carbon matrix. For the catalyst presented in Fig. S2a† no melamine was added in the preparation process, and there was still obvious pore formation. However, compared with several other catalysts, the pore size of the cavity was significantly smaller, indicating that the porous characteristics of γ-CD-MOF were maintained to a certain extent during pyrolysis. When the melamine content is lower or higher than the doping amount of FePc@CD/M (1 : 20)-1000, the pores of carbon materials rarely form cross-linked porous structures. The formation of the internal cavity also stems from the escape of the gas formed by the decomposition of melamine. Upon changing the pyrolysis temperature, no obvious change was observed from the local TEM. As for FePc@CD/M (1 : 20)-1000, only unobvious pores can be observed inside the material surface. It is speculated that most of the pores collapse and the pores are buried due to the high reaction temperature. Fig. S3† shows the SEM images of different catalysts. The catalysts prepared by pyrolysis at 800 °C and 900 °C have relatively smooth surfaces. With the increase of temperature, the catalyst surface becomes rougher and an obvious pore structure appears. The pore structure of the catalyst prepared at 1100 °C mostly collapsed due to the excessively high reaction temperature, and the carbon particles also agglomerated to form agglomerates, indicating that the appropriate heating temperature is an important factor in obtaining a hierarchical porous structure. At the same time, the reason why a large number of particles appear on the surface of the material is that a large amount of potassium in the γ-CD-MOF skeleton precipitates on the surface at high temperature. It is not only useless for catalysis, but also clogs pores and conceals active sites.

Fig. 1c shows the high-angle annular dark-field scanning transmission electron microscope (HAADF-STEM) image, displaying the atomic structure of the FePc@CD/M (1 : 20)-1000 catalyst. The large amounts of isolated bright dots can be attributed to heavier Fe atoms due to the difference in the *Z*-contrast of C, N and Fe, indicating that Fe is well-dispersed on the carbon nanosheets.^29,30^ No noticeable metal aggregation was observed in the HRTEM image (Fig. 1d), indicating that there was no formation of Fe nanoparticles on the carbon matrix. The Energy-Dispersive X-ray (EDX) elemental mapping images revealed that N, O and Fe atoms are uniformly dispersed throughout the entirety of the carbon nanosheets (Fig. 1e–h).

**Fig. 2:**
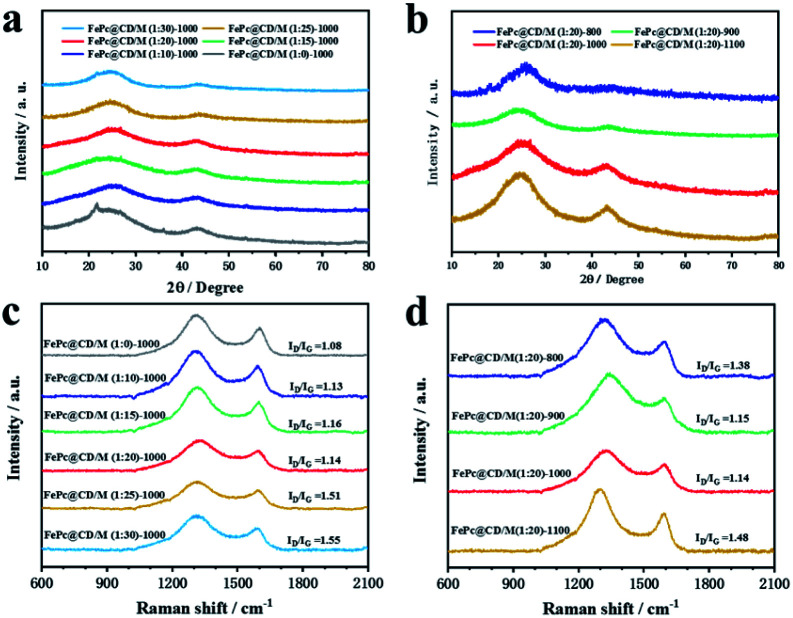
Structural and compositional examinations of the as-prepared samples. (a and b) XRD patterns and (c and d) Raman spectra.

XRD was used to characterize the chemical phase and crystal structure of the samples. As displayed in Fig. 2a and b, the XRD patterns of all the samples exhibited two typical broad peaks at around 25.5° and 44.2°, corresponding to the (002) and (101) crystal phases of graphitic carbon, respectively. For the samples doped with different proportions of melamine (Fig. 2a) and prepared at different pyrolysis temperatures (Fig. 2b), no other diffraction peaks were observed in any of the samples, indicating that there was no crystalline metal or metal compound in the prepared catalyst. The Raman spectra of the catalysts in Fig. 2c and d exhibit two well-defined peaks: the D band at 1319 cm^−1^ corresponds to graphitic carbon, and the G band at 1592 cm^−1^ corresponds to disordered carbon and defects. The D band is obviously higher than the G band, which means that there are many defects in the carbon structure and the graphitization degree is low. The *I*_D_/*I*_G_ of catalysts doped with different proportions of melamine (with the increase of melamine content, the *I*_D_/*I*_G_ values are 1.13, 1.14, 1.16, 1.51, and 1.55, respectively) was significantly higher than that of the undoped catalyst (1.08), indicating that under the same pyrolysis conditions, additional N doping will lead to a surface carbon atom rearrangement and more defects. The Raman results of catalysts prepared at different pyrolysis temperatures showed that high pyrolysis temperature was not conducive to the graphitization of samples (with the increase of temperature, the *I*_D_/*I*_G_ values are 1.38, 1.15, 1.14, and 1.48, respectively). Among all the samples doped with melamine, FePc@CD/M (1 : 20)-1000 has the lowest *I*_D_/*I*_G_, which indicates the highest degree of graphitization.^31,32^ Even though FePc@CD/M (1 : 20)-1000 has the highest degree of graphitization, its HRTEM image (Fig. S10†) shows no lattice streaks of graphitized carbon and curved disordered primary crystalline carbon can be seen in the red circles.

In order to study the porous structure and specific surface area of the prepared materials, nitrogen adsorption–desorption curves were recorded. Fig. S4† shows the nitrogen adsorption–desorption curves and pore size distributions of the prepared catalysts. The Brunauer–Emmett–Teller (BET) specific surface area and pore volume of all samples are shown in Table S1.† In general, the main factors affecting the specific surface area of the catalysts are the porous characteristics of MOF itself, the new pore structure formed due to the escape of gas during pyrolysis, and the collapse of the original cavity structure caused by high temperature. The pore size distribution of all samples was concentrated in the range of 1.5–10 nm, indicating the existence of a mesoporous structure. It is obvious that the doping of melamine and the change of pyrolysis temperature can indeed regulate the surface area. This is because, in the pyrolysis process, with the gradual increase of temperature, when the temperature reaches about 350 °C, melamine begins to decompose into ammonia and hydrogen cyanide gas escapes, which can form a large number of pores on the surface of the material in the process of gas escape. However, as the subsequent pyrolysis continues, these channels will collapse at too high temperature, which will lead to the decrease of specific surface area. In addition, there is a large amount of K in the γ-CD-MOF framework. When the temperature reaches the boiling point of K (759 °C), some K will evaporate, which on the other hand leads to the formation of pores and the loss of specific surface area due to the destruction of the original framework. FePc@CD/M (1 : 20)-1000 has a large specific surface area (1055.317 m^2^ g^−1^) and hierarchical pore structure.

**Fig. 3:**
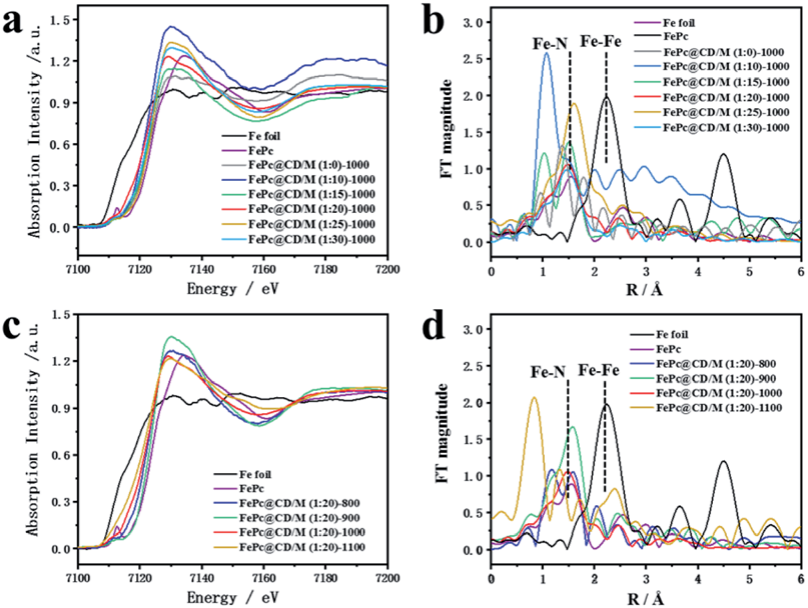
(a and c) Fe K-edge EXAFS spectra and (b and d) XANES spectra of all samples, FePc and Fe foil.

Fig. 3 shows the XANES and EXAFS measurements to determine the chemical state and coordination environment at the atomic level. Compared with the XANES curves of Fe foil and FePc samples, the absorption edges of all the prepared catalysts are located between the absorption edges of Fe and FePc samples and are close to the absorption edges of FePc (Fig. 3a and c). Since the valence state of the measured element can be reflected by the absorption edge of XANES, the average valence state of Fe species in the prepared sample should be similar to the average valence state of Fe species in FePc.^33,34^ The EXAFS curves of Fe foil, FePc and the prepared samples are shown in Fig. 3b and d. For the curve of Fe foil and FePc@CD/M (1 : 20)-1000, the peak at 2.2 Å is attributed to the Fe–Fe bond. Other samples show a main peak near 1.50 Å, which may be attributed to the Fe–N scattering path, and there is no obvious Fe–Fe peak (2.2 Å), which also implies that there is no agglomeration between Fe atoms.^33,35^

**Fig. 4:**
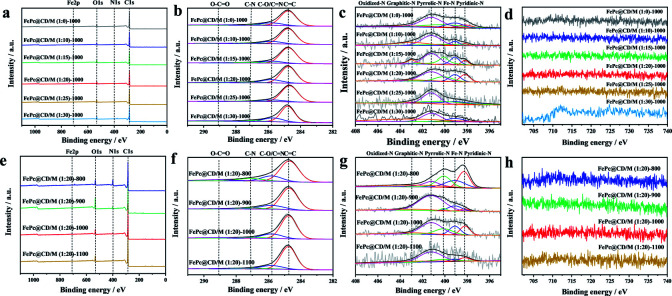
(a and e) XPS survey spectra, (b and f) C 1s region, (c and g) N 1s region, and (d and h) Fe 2p region of all samples.

We further studied the surface chemical state of the prepared catalysts by XPS (Fig. 4). The spectra show the presence of C, N, Fe, and O. The high-resolution C_1s_ spectra in Fig. 4b and f were decomposed into four sub-peaks, C=C (284.7 eV), C–O/C=N (285.8 eV), C–N (287.1 eV) and O–C=O (289.2 eV).^36^ Their relative contents are shown in Table S2.† Due to the low iron content and high dispersion, the iron spectrum cannot be clearly distinguished.^37,38^ However, the content of active Fe–N_*x*_ species can be obtained from the nitrogen spectrum. The N_1s_ peaks in the spectra (Fig. 4c and g) can be divided into five N types, pyridinic-N (398.2 eV), Fe–N (399.1 eV), pyrrolic-N (400.1 eV), graphitic-N (401.2 eV) and oxidized-N (402.9 eV),^39–41^ and their relative content is shown in Table S3.† With the increase of the doping amount of melamine, the total content of N in the catalyst is 2.71%, 1.84%, 1.88%, 4.39%, 2.40% and 2.22%, respectively, which increases first and then decreases. FePc@CD/M (1 : 20)-1000 has the highest relative content of N (4.39%), and the proportion of the sum of the relative content of Fe–N and pyridinic-N is also the highest (31.37%). It is observed that the content of graphitic-N in the FePc@CD/M (1 : 0)-1000 catalyst without melamine doping is much higher than that in other catalysts. This fully illustrates that the doping of melamine does increase the defects in the material and reduce the graphitization degree of the material, which is confirmed by the Raman test results above. With the increase of pyrolysis temperature, the total content of N in the catalyst was 11.14%, 3.82%, 4.39%, and 1.57%, respectively. At the same time, the proportion of the sum of the relative content of Fe–N and pyridinic-N (43.59%, 17.00%, 31.37% and 9.63%) also has the same changing law. According to the above analysis of HAADF-STEM, XPS and EXAFS measurements, the Fe atoms in the FePc@CD/M (1 : 20)-1000 samples were highly dispersed in the N-doped porous carbon layer.

**Fig. 5:**
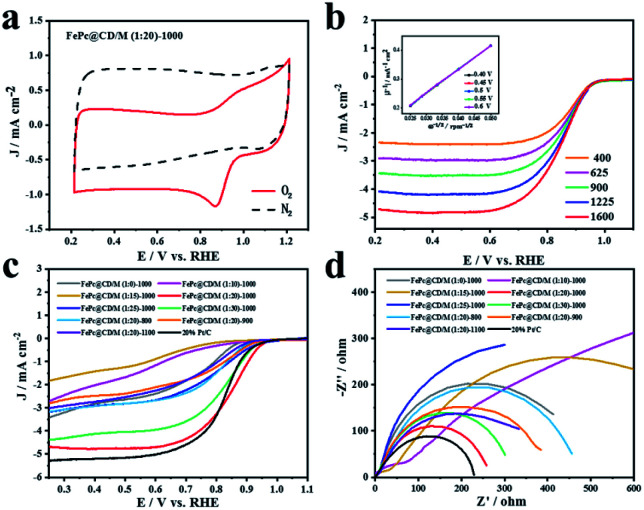
(a) The cyclic voltammetry curves of FePc@CD/M (1 : 20)-1000, (b) the LSV curves of FePc@CD/M (1 : 20)-1000 in 0.1 M KOH (inset: K–L plots of FePc@CD/M (1 : 20)-1000 at various potentials), (c) LSV curves of all samples and 20% Pt/C at a fixed rotation rate, and (d) Nyquist plots of all samples and 20% Pt/C.

The ORR activity of the FePc@CD/M (1 : 20)-1000 catalyst was studied by cyclic voltammetry (CV) and linear sweep voltammetry (LSV) in O_2_- or N_2_-saturated alkaline solution (0.1 M KOH). In N_2_-saturated electrolyte, no obvious slope was observed for FePc@CD/M (1 : 20)-1000. In contrast, FePc@CD/M (1 : 20)-1000 showed a significant cathodic peak at 0.882 V (*vs.* RHE) in O_2_-saturated solution, indicating that FePc@CD/M (1 : 20)-1000 had excellent ORR activity (Fig. 5a). In order to understand the kinetics of the catalyst for the ORR, LSV curves were recorded at different rotational speeds (Fig. 5b). With the increase of mechanical rotational speed, the mass transfer rate and diffusion rate also increase, which will inevitably lead to a typical increase in current density. The Koutecky–Levich (K–L) diagram shows that FePc@CD/M (1 : 20)-1000 follows the main four-electron transfer pathway (3.84).^42^ The LSV polarization curves of all samples and a commercial 20% Pt/C catalyst were obtained (Fig. 5c). It should be noted that the contents of graphitic-N, pyridinic-N and Fe–N_*x*_ are closely related to ORR activity. The uniform distribution of N and O changed the surface electron distribution of carbon materials, which was more conducive to the adsorption of O_2_, reduced the O–O bond breaking barrier, and adsorbed the intermediates in the oxygen reduction reaction chemically and physically on the carbon surface. At the same time, their presence can adjust the electronic structure of the catalyst and promote the catalytic activity of the ORR. It can be concluded that among the synthesized catalysts, FePc@CD/M (1 : 20)-1000 exhibits an optimal catalytic performance (*E*_onset_ = 0.9886 V, *E*_1/2_ = 0.846 V *vs.* RHE) compared with 20% Pt/C (*E*_onset_ = 0.9846, *E*_1/2_ = 0.8219 *vs.* RHE). These results are superior to those of most relevant catalysts, as shown in Table S4.† This is consistent with the results of XPS, highlighting its good catalytic performance, high content of pyridinic-N, graphitic-N and Fe–N, and large specific surface area that cannot be ignored. However, compared with 20% Pt/C, FePc@CD/M (1 : 20)-1000 does not have a higher current density (Table S5†), which is attributed to its low graphitization degree, and is not conducive to the transfer of electrons, as shown in the Raman results. The slope of the Tafel curve is inversely proportional to the ORR electron transfer coefficient, and is used as an important parameter to evaluate the electrode reactivity. The Nyquist plots of the catalysts are shown in Fig. 5d, which were fitted by the equivalent circuit (Fig. S5 and S6†), and the fitting results are shown in Table S6.†

At the interface of the electrode, catalyst and electrolyte, electrons in the electrode react with ions in the electrolyte as charge carriers. At the interface between the electrode and electrolyte, the electrode reaction due to charge transfer is closely related to the physical conditions and physical properties of the interface. The smaller the impedance is, the smaller the resistance at the solid–liquid interface to the charge transfer, the easier the charge transfer, and the more conducive it is to the ORR process.^43–45^ Comparing the catalyst impedance *R*_p_, FePc@CD/M (1 : 20)-1000 had the lowest impedance among the prepared catalysts, which was close to that of commercial 20% Pt/C. The impedance of the catalysts at different pyrolysis temperatures is similar to *I*_D_/*I*_G_, so the defect degree and graphitization degree of the material may be the main factors affecting the impedance.^46,47^ From the fitting results of the equivalent circuit, FePc@CD/M (1 : 10)-1000 and FePc@CD/M (1 : 15)-1000 exhibit two-step two-electron processes, and the second impedance step is greater than the first step. The capacitance (*C*) of the catalyst fitted by Nyquist plots is shown in Table S6.† The larger the capacitance value, the higher the surface roughness of the material, and the larger the real active area. The pores and internal cavities on the surface of the carbon material provide a large specific surface area, which can shorten the mass transfer path, promote electrolyte penetration, and promote contact between the active site and O_2_. In addition, the electrochemically active surface area (ECSA) was evaluated, which was proportional to the electrochemical double-layer capacitance (*C*_dl_) obtained by CV measurement at different scanning rates (Fig. S7 and S8†). ECSA is related to the real active area of the catalyst. The high density of catalytic sites and the faster oxygen diffusion rate are conducive to rapid mass transfer and oxygen transfer in the ORR. The results obtained by this method are partially different from those obtained by impedance fitting above. The FePc@CD/M (1 : 20)-1000 catalyst with the best ORR activity shows the smallest ESCA, indicating that it has a small effective contact area with the reactants in the catalytic reaction, and this proves that the catalyst has excellent catalytic performance.

**Fig. 6:**
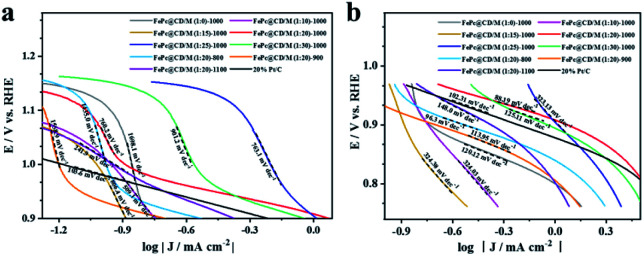
Tafel plots of all samples and 20% Pt/C: (a) mixed diffusion–kinetic limitation region, (b) Tafel region.

Then, the Tafel curve obtained by fitting the polarization curve in Fig. 5c is used to explore the reaction kinetics (Fig. S9†). Overall, it can be divided into three linear regions, namely electrochemically controlled region (low overpotential region), mixed diffusion–kinetic limitation region (high overpotential region) and diffusion-controlled region. The rate-determining step of the ORR is the electrochemically controlled step (Fig. 6a). The slope of the Tafel curve of FePc@CD/M (1 : 20)-1000 is 88.19 mV dec^−1^ in the mixed control region (Fig. 6b), which is the lowest among all synthesized catalyst materials, even lower than that of the commercial 20% Pt/C catalyst (102.31 mV dec^−1^). It shows that its overpotential is lower than that of 20% Pt/C, and it has better reaction kinetics.

**Fig. 7:**
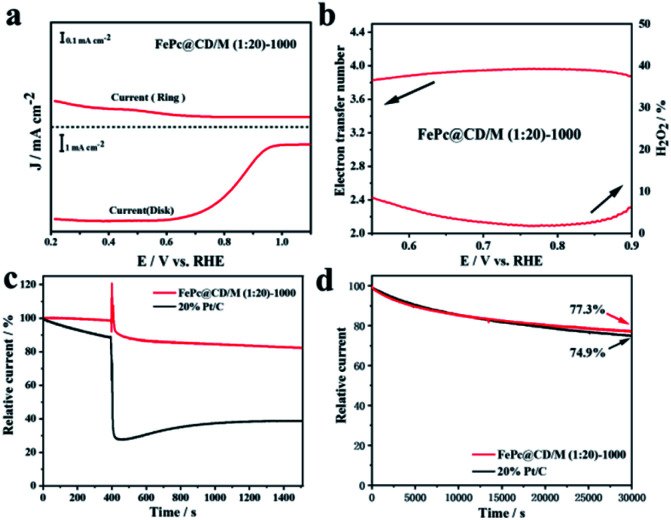
(a) The RRDE measurements recorded with a scan rate of 5 mV s^−1^ and a rotation rate of 1600 rpm, (b) electron-transfer number of FePc@CD/M (1 : 20)-1000 and the amount of hydrogen peroxide produced, (c) tolerance to methanol crossover of FePc@CD/M (1 : 20)-1000 and 20% Pt/C with addition of 3 M methanol at 400 s and (d) stability evaluation of FePc@CD/M (1 : 20)-1000 and 20% Pt/C.

To further study the electron transfer number (*n*) and peroxide yield (H_2_O_2_%) of the synthesized samples, the RRDE measurements of FePc@CD/M (1 : 20)-1000 recorded by the rotating ring-disk electrode (RRDE) are shown in Fig. 7a and b. The electron transfer number (*n*) of FePc@CD/M (1 : 20)-1000 was higher than 3.8 from 0.5 V to 0.9 V (*vs.* RHE), and the corresponding H_2_O_2_ yield (%) was less than 8%, which was consistent with the results of the above K–L curve.^48^ These results showed that the FePc-derived catalysts exhibited good performance for the ORR under alkaline conditions. In addition, possible cross-effects caused by small organic molecules such as methanol were tested (Fig. 7c). After the addition of methanol (final concentration of 3 M) at 400 s, FePc@CD/M (1 : 20)-1000 recovered rapidly and the attenuation was very small. The reason for the very small current decay of FePc@CD/M (1 : 20)-1000 is that after the addition of methanol to the electrolyte, the methanol undergoes an oxidation reaction and the oxidation products adhere to the catalyst surface, affecting the contact between the catalyst and oxygen, resulting in the reaction not proceeding properly. In contrast, for the Pt/C catalyst, the addition of methanol triggered a sharp increase in current density, which was difficult to recover from, indicating that its tolerance to chemical corrosion was much weaker than that of FePc@CD/M (1 : 20)-1000. The current of the Pt/C catalyst decreases after the addition of methanol for two main reasons: firstly, the oxidation product of methanol covers the surface of Pt/C, making it difficult for the Pt/C to come into contact with oxygen resulting in a lower current. Secondly, the Pt/C catalyzes the oxidation of methanol creating a mixed potential resulting in a lower ORR current. The durability of FePc@CD/M (1 : 20)-1000 was also evaluated, as shown in Fig. 7d. Compared with 20% Pt/C (74.9%), FePc@CD/M (1 : 20)-1000 can maintain a slightly larger original current (77.3%) after continuous operation for 30 000 s. Comparing the TEM images (Fig. S11†) before and after the CA test, we found that the interconnected cavities did not change before and after the durability test.

## 4. Conclusions

Due to the adaptation of the two molecules in molecular size, γ-CD-MOF can encapsulate a single FePc molecule in the internal cavity to form the host–guest FePc@γ-CD-MOF structure. The Fe/N/C catalyst was prepared by pyrolysis of this precursor doped with melamine. Due to the restriction effect of the host and guest at the molecular level, the stacking of FePc was inhibited, and the agglomeration of Fe centers during pyrolysis was prevented. The atoms were dispersed on the carbon matrix in the form of Fe–N_*x*_ active sites, which was confirmed by the results of HAADF-STEM and XANES experiments. In addition, the catalyst showed excellent ORR activity and good methanol tolerance. Our work provides a simple and feasible way to design non-noble metal catalysts with high atom utilization, high performance and high atom dispersion.

## Conflicts of interest

There are no conflicts to declare.

## Supplementary Material

NA-004-D2NA00095D-s001
