# Supplementary material for: The performance of an atomically dispersed oxygen reduction catalyst prepared by γ-CD-MOF integration with FePc
Source: Nanoscale Adv. 2022 Apr 11;4(9):2171–9. doi: 10.1039/d2na00095d (PMC9419513; doi:10.1039/d2na00095d)
Supplement: NA-004-D2NA00095D-s001 [file NA-004-D2NA00095D-s001.pdf]

Electronic Supplementary Material (ESI) for Nanoscale Advances.
This journal is © The Royal Society of Chemistry 2022

Supporting Information

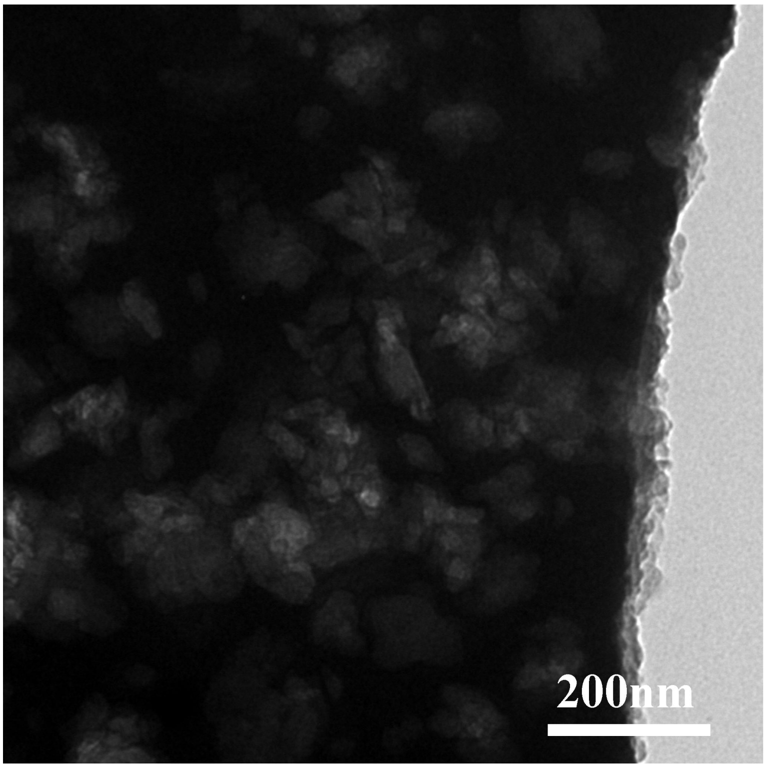

Fig. S1 TEM image of FePc@CD/M (1:20)-1000.

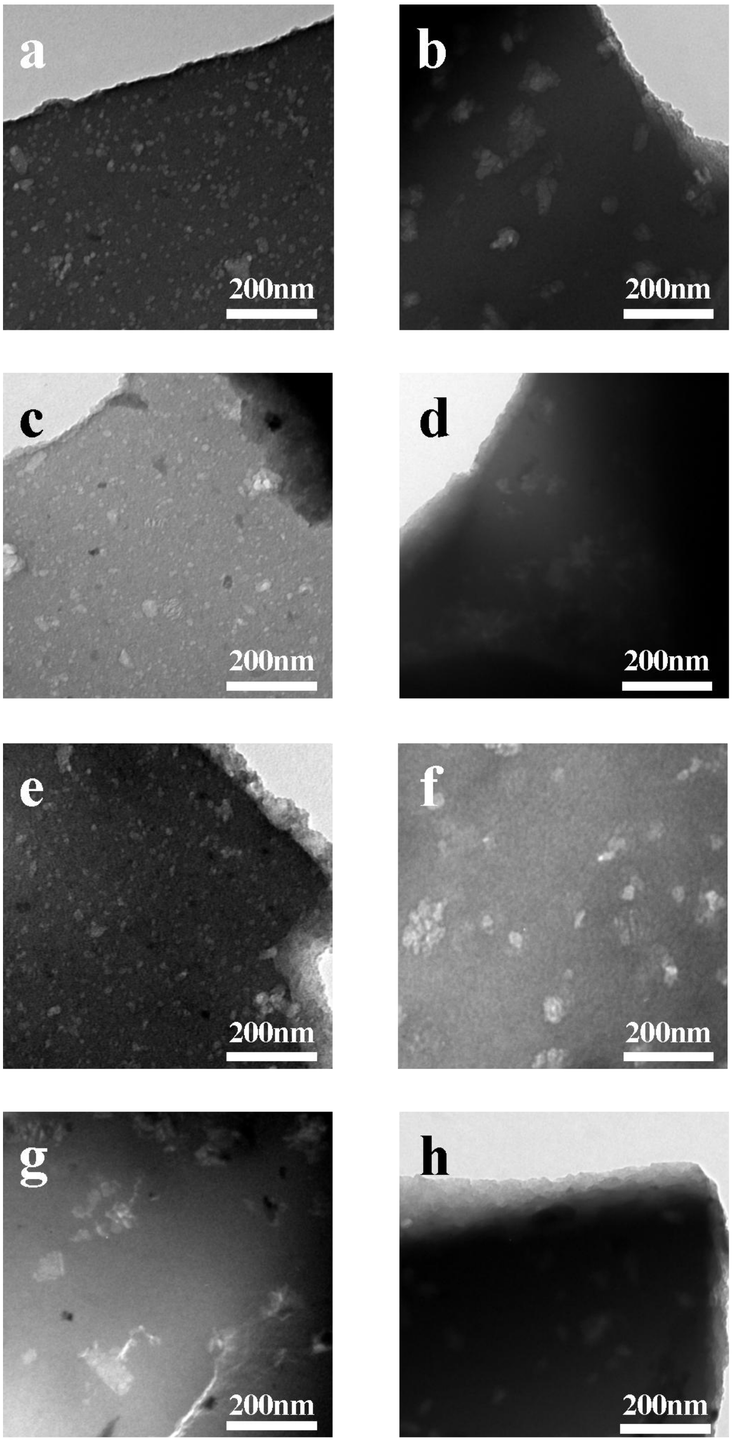

Fig. S2 **TEM image of** a) FePc@CD/M (1:0)-1000, b) FePc@CD/M (1:10)-1000, c) FePc@CD/M (1:15)-1000, d) FePc@CD/M (1:25)-1000, e) FePc@CD/M (1:30)-1000, f) FePc@CD/M (1:20)-800, g) FePc@CD/M (1:20)-900, h) FePc@CD/M (1:20)-1100.

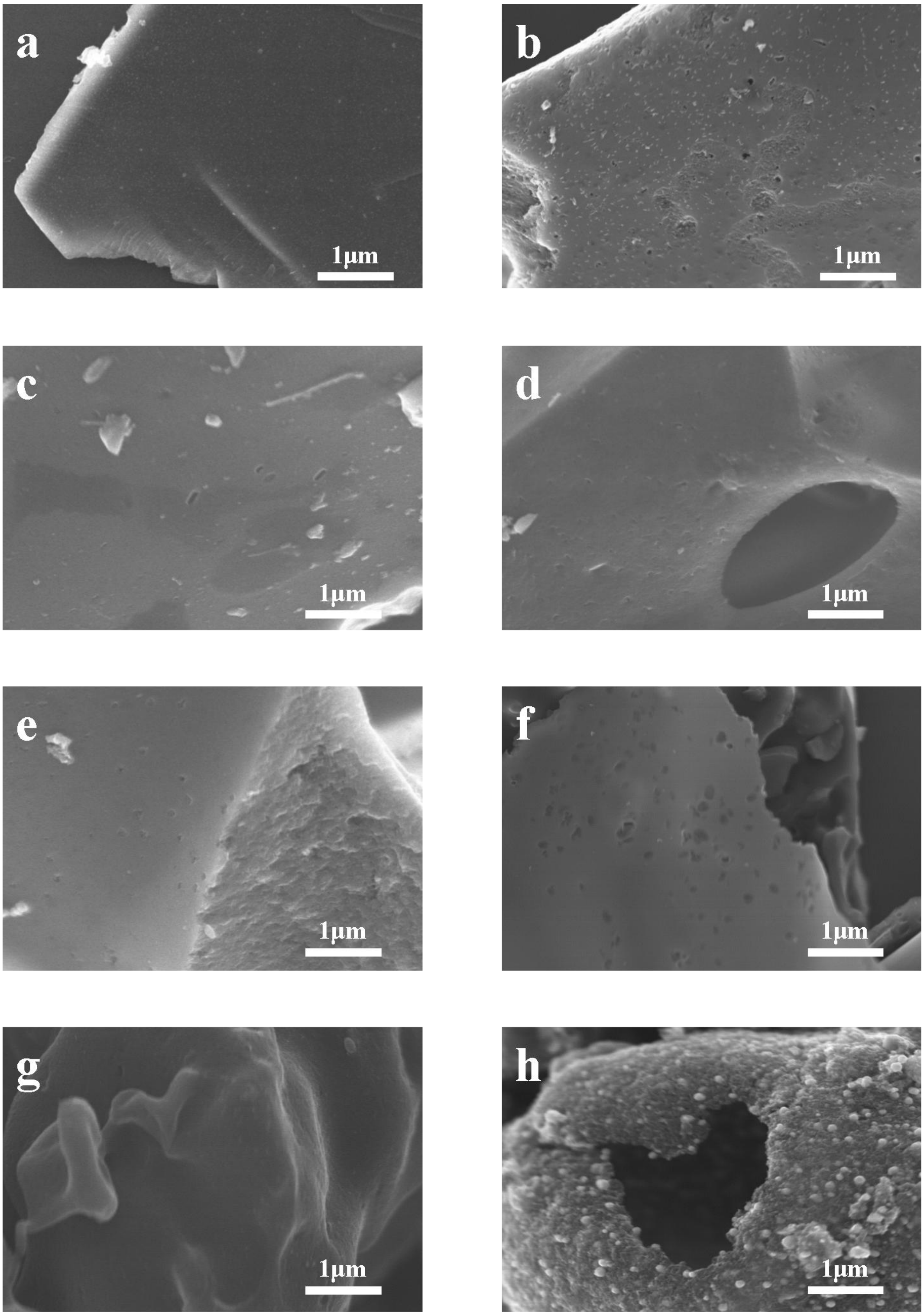

Fig. S3 **SEM image of** a) FePc@CD/M (1:0)-1000, b) FePc@CD/M (1:10)-1000, c) FePc@CD/M (1:15)-1000, d) FePc@CD/M (1:25)-1000, e) FePc@CD/M (1:30)-1000, f) FePc@CD/M (1:20)-800, g) FePc@CD/M (1:20)-900, h) FePc@CD/M (1:20)-1100.

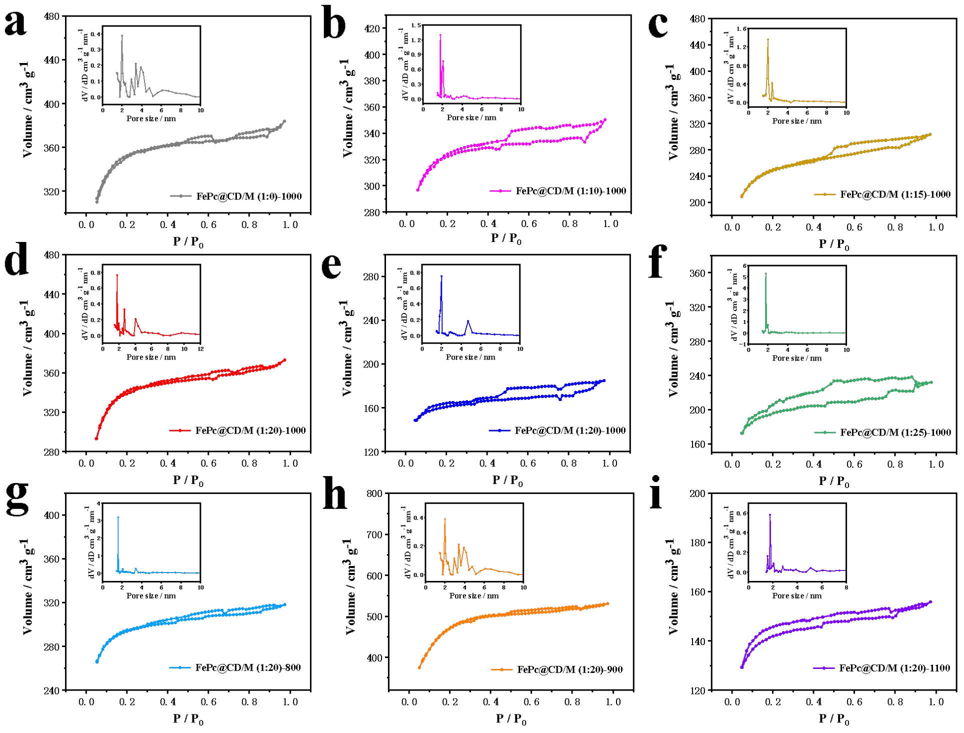

Fig. S4 N_2 adsorption−desorption curves of all catalysts.

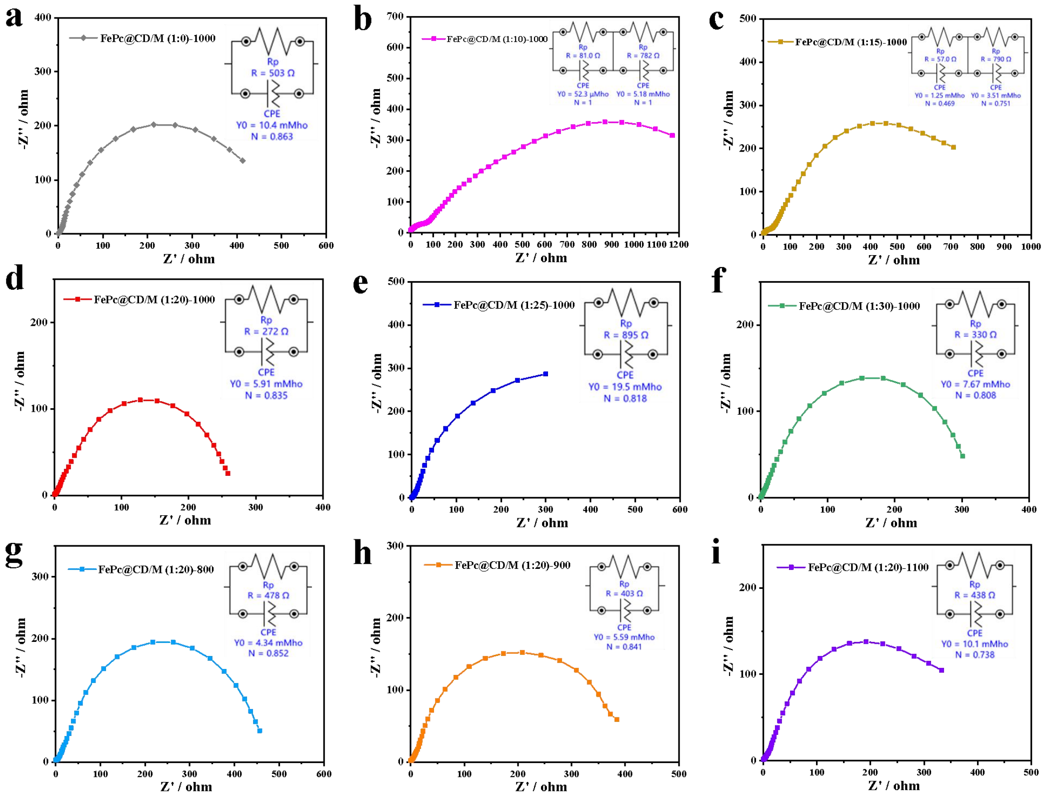

Fig. S5 The Nyquist diagram and equivalent circuit of a) FePc@CD/M (1:0)-1000, b) FePc@CD/M (1:10)-1000, c) FePc@CD/M (1:15)-1000, d) FePc@CD/M (1:20)-1000, e) FePc@CD/M (1:25)-1000, f) FePc@CD/M (1:30)-1000, g) FePc@CD/M (1:20)-800, h) FePc@CD/M (1:20)-900, i) FePc@CD/M (1:20)-1100.

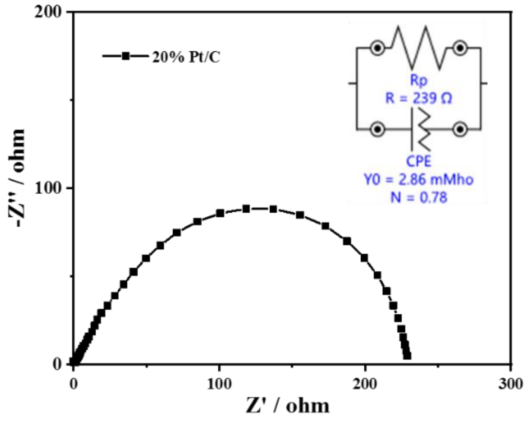

Fig. S6 The Nyquist diagram and equivalent circuit of 20% Pt/C.

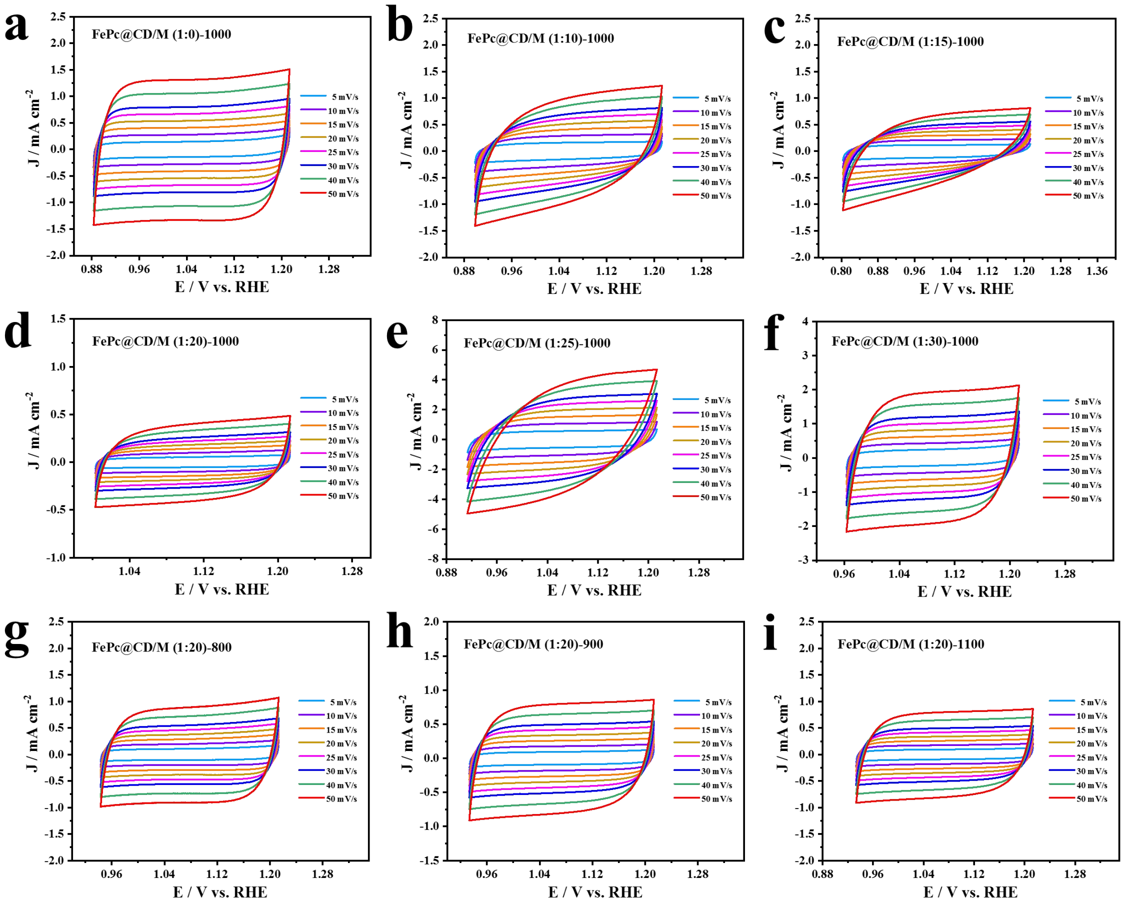

Fig. S7 Cyclic voltammograms in the region without faradaic processes with different scan rates of a) FePc@CD/M (1:0)-1000, b) FePc@CD/M (1:10)-1000, c) FePc@CD/M (1:15)-1000, d) FePc@CD/M (1:20)-1000, e) FePc@CD/M (1:25)-1000, f) FePc@CD/M (1:30)-1000, g) FePc@CD/M (1:20)-800, h) FePc@CD/M (1:20)-900, i)FePc@CD/M (1:20)-1100.

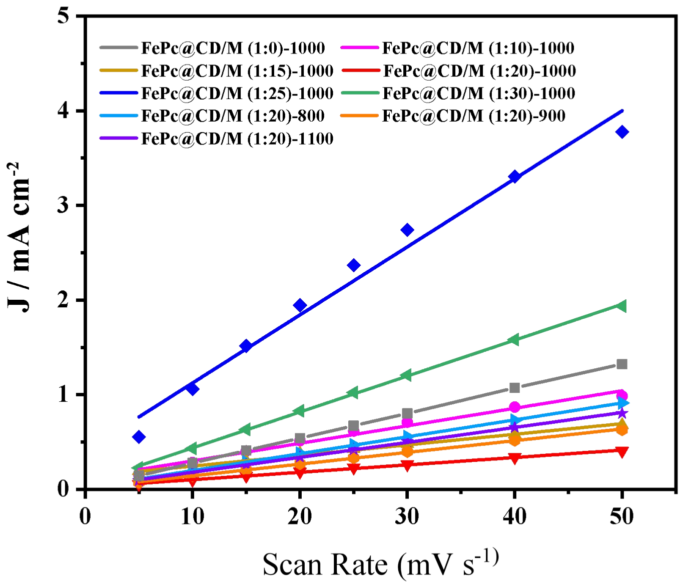

Fig. S8 Capacitive currents as a function of the scan rate of all samples.

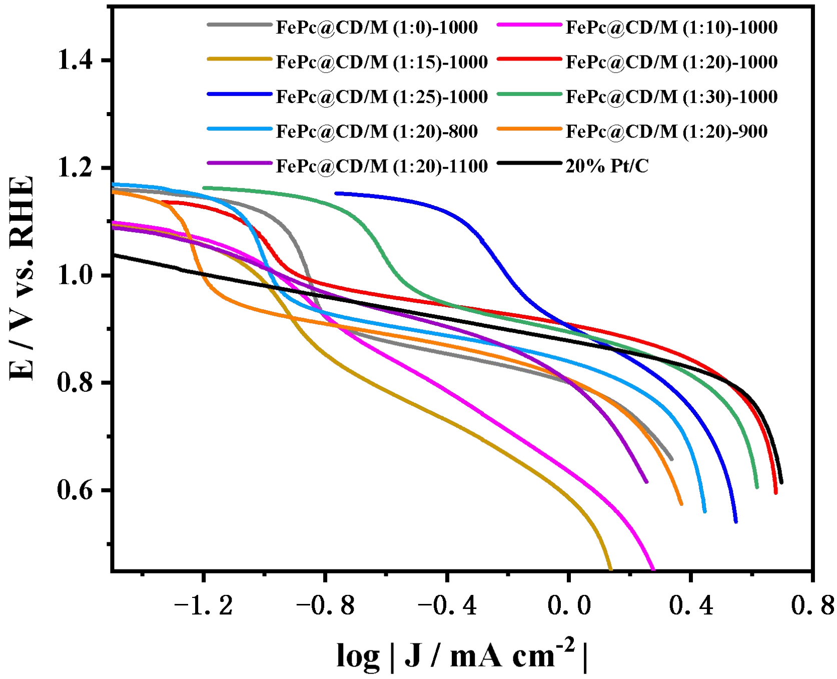

Fig. S9 Tafel plots of all samples and 20% Pt/C.

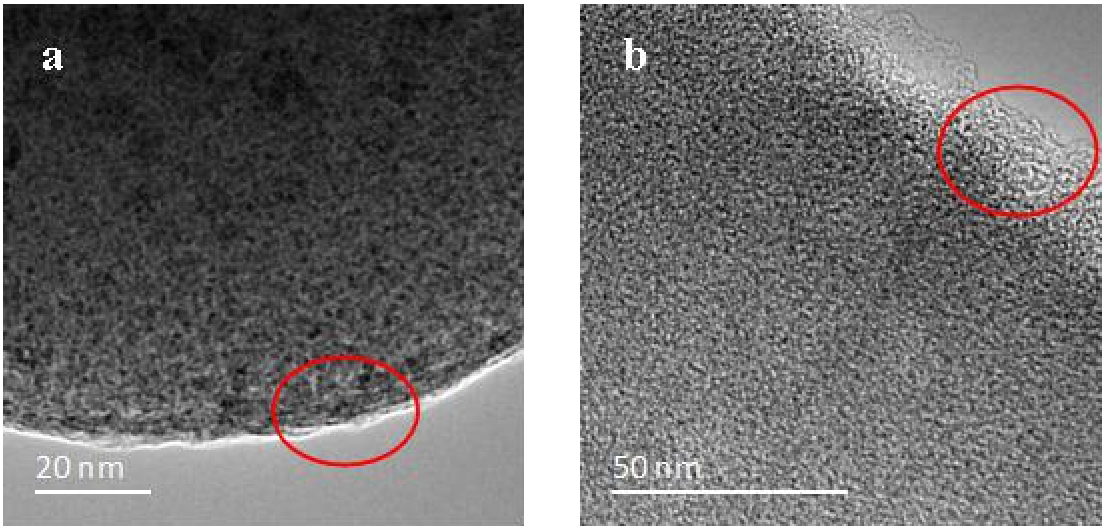

Fig. S10 (a) and (b)HRTEM image of FePc@CD/M (1:20)-1000.

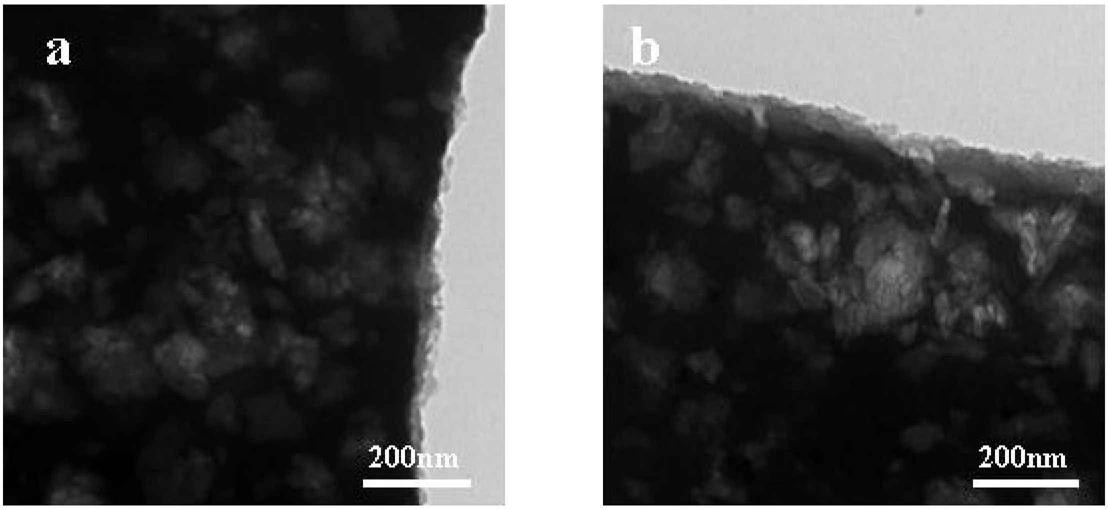

Fig. S11 (a)TEM image of FePc@CD/M (1:20)-1000 before CA test. (b)TEM image of FePc@CD/M (1:20)-1000 after CA test.

Table S1. The BET surface area and pore volume of all samples.

**Sample**	**BET surface area (m^2g^{-1})**	**Micropore volume (cm^3g^{-1})**
FePc@CD/M (1:0)-1000	492.599	0.068
FePc@CD/M (1:10)-1000	977.297	0.054
FePc@CD/M (1:15)-1000	786.376	0.106
FePc@CD/M (1:20)-1000	1055.317	0.060
FePc@CD/M (1:25)-1000	645.412	0.049
FePc@CD/M (1:30)-1000	608.612	0.077
FePc@CD/M (1:20)-800	902.359	0.049
FePc@CD/M (1:20)-900	1510.607	0.093
FePc@CD/M (1:20)-1100	435.745	0.023

Table S2. Deconvolution of C1s XPS spectra

Sample	C=C	C-O/C=N	C-N	O-C=C
FePc@CD/M (1:0)-1000	72.72	16.42	4.04	6.82
FePc@CD/M (1:10)-1000	77.66	15.34	4.57	2.43
FePc@CD/M (1:15)-1000	80.08	12.86	5.79	1.27
FePc@CD/M (1:20)-1000	72.84	18.13	4.88	4.16
FePc@CD/M (1:25)-1000	82.59	12.62	4.12	0.68
FePc@CD/M (1:30)-1000	65.63	21.23	7.28	5.86
FePc@CD/M (1:20)-800	64.23	17.20	9.94	8.63
FePc@CD/M (1:20)-900	74.16	17.84	5.89	2.11
FePc@CD/M (1:20)-1100	75.46	16.02	6.14	2.38

Table S3. Deconvolution of N1s XPS spectra

Sample	N content (at%)	Pyridinic-N (%)	Fe−N (%)	pyrrolic-N (%)	graphitic-N (%)	oxidized-N (%)
FePc@CD/M (1:0)-1000	2.71	12.41	0.00	41.01	31.90	14.68
FePc@CD/M (1:10)-1000	1.84	3.30	9.83	15.52	39.70	31.65
FePc@CD/M (1:15)-1000	1.88	7.47	17.54	23.96	40.20	10.82
FePc@CD/M (1:20)-1000	4.39	15.01	16.36	18.15	41.82	8.66
FePc@CD/M (1:25)-1000	2.4	5.11	25.90	30.29	36.35	2.35
FePc@CD/M (1:30)-1000	2.22	4.34	13.89	2.84	36.79	42.13
FePc@CD/M (1:20)-800	11.14	24.47	19.12	23.69	16.99	15.72
FePc@CD/M (1:20)-900	3.82	3.78	13.22	2.77	71.18	9.06
FePc@CD/M (1:20)-1100	1.57	6.02	3.61	8.65	33.35	48.37

Table S4 Summary of performance for ORR in 0.1 M KOH.

Catalysts	E_{onset} (V vs. RHE)	$E_{1/2}$ (Vs. RHE)	Reference
**FePc@CD/M (1:20)-1000**	**0.988**	**0.846**	**This work**
Fe-N-C-900	0.96	0.85	[1]
Fe/OES	1.0	0.85	[2]
Fe-NCCs	/	0.82	[3]
Fe–N/C catalyst	0.923	0.81	[4]
L-FeNC	/	0.89	[5]
Fe–N/C-SAC	0.89	/	[6]
Fe-ISAs/CN	0.9	/	[7]
Fe-N-SCCFs	1.03	0.883	[8]
Fe SA-NSC-900	0.96	0.86	[9]

Table S5 Detailed data of electrocatalytic properties for the all as-prepared samples compared with 20% Pt/C.

Sample	E_{onset} (V vs.RHE)	$E_{1/2}$ (V vs.RHE)	| J | (mA cm^{-2})
FePc@CD/M (1:0)-1000	0.912	0.812	3.686
FePc@CD/M (1:10)-1000	0.832	/	2.983
FePc@CD/M (1:15)-1000	0.893	/	1.987
FePc@CD/M (1:20)-1000	0.988	0.846	4.728
FePc@CD/M (1:25)-1000	0.922	0.833	3.348
FePc@CD/M (1:30)-1000	0.962	0.833	4.608
FePc@CD/M (1:20)-800	0.967	0.818	3.305
FePc@CD/M (1:20)-900	0.976	0.812	2.931
FePc@CD/M (1:20)-1100	0.928	/	3.099
20% Pt/C	0.985	0.822	5.323

Table S6 The fitting results of Nyquist diagrams

Sample	Rp (Mho)	Y0 (mMho)	N	C (mF)
FePc@CD/M (1:0)-1000	503	10.4	0.863	11.2
FePc@CD/M (1:10)-1000	(Rp^1) 81.0	($Y0^1$) 0.0523	1	0.0523
	(Rp^2) 782	($Y0^2$) 5.18	1	5.18
FePc@CD/M (1:15)-1000	(Rp^1) 57	($Y0^1$) 1.25	0.469	0.0628
	(Rp^2) 790	($Y0^2$) 3.51	0.751	4.92
FePc@CD/M (1:20)-1000	272	5.91	0.835	6.49
FePc@CD/M (1:25)-800	895	19.5	0.818	36.8
FePc@CD/M (1:30)-900	330	7.67	0.808	9.53
FePc@CD/M (1:20)-800	478	4.34	0.852	4.93
FePc@CD/M (1:20)-900	403	5.59	0.841	6.52
FePc@CD/M (1:20)-1100	438	10.1	0.738	17.1
20% Pt/C	239	2.86	0.78	2.55

References

1. He, Q.; Meng, Y.; Zhang, H.; Zhang, Y.; Sun, Q.; Gan, T.; Xiao, H.; He, X.; Ji, H., Amino-metalloporphyrin polymers derived Fe single atom catalysts for highly efficient oxygen reduction reaction. *Science China Chemistry* **2020,** *63* (6), 810-817.

2. Hou, C. C.; Zou, L.; Sun, L.; Zhang, K.; Liu, Z.; Li, Y.; Li, C.; Zou, R.; Yu, J.; Xu, Q., Single-Atom Iron Catalysts on Overhang-Eave Carbon Cages for High-Performance Oxygen Reduction Reaction. *Angew Chem Int Ed Engl* **2020,** *59* (19), 7384-7389.

3. Jia, N.; Xu, Q.; Zhao, F.; Gao, H.-X.; Song, J.; Chen, P.; An, Z.; Chen, X.; Chen, Y., Fe/N Codoped Carbon Nanocages with Single-Atom Feature as Efficient Oxygen Reduction Reaction Electrocatalyst. *ACS Applied Energy Materials* **2018,** *1* (9), 4982-4990.

4. Lin, L.; Zhu, Q.; Xu, A. W., Noble-metal-free Fe-N/C catalyst for highly efficient oxygen reduction reaction under both alkaline and acidic conditions. *J Am Chem Soc* **2014,** *136* (31), 11027-33.

5. Jiang, X.; Chen, J.; Lyu, F.; Cheng, C.; Zhong, Q.; Wang, X.; Mahsud, A.; Zhang, L.; Zhang, Q., In situ surface-confined fabrication of single atomic Fe-N4 on N-doped carbon nanoleaves for oxygen reduction reaction. *Journal of Energy Chemistry* **2021,** *59*, 482-491.

6. Wu, D.; Liu, W.; Hu, J.; Zhu, C.; Jing, H.; Zhang, J.; Hao, C.; Shi, Y., Direct transformation of raw biomass into a Fe–Nx–C single-atom catalyst for efficient oxygen reduction reaction. *Materials Chemistry Frontiers* **2021,** *5* (7), 3093-3098.

7. Chen, Y.; Ji, S.; Wang, Y.; Dong, J.; Chen, W.; Li, Z.; Shen, R.; Zheng, L.; Zhuang, Z.; Wang, D.; Li, Y., Isolated Single Iron Atoms Anchored on N-Doped Porous Carbon as an Efficient Electrocatalyst for the Oxygen Reduction Reaction. *Angew Chem Int Ed Engl* **2017,** *56* (24), 6937-6941.

8. Wang, B.; Wang, X.; Zou, J.; Yan, Y.; Xie, S.; Hu, G.; Li, Y.; Dong, A., Simple-Cubic Carbon Frameworks with Atomically Dispersed Iron Dopants toward High-Efficiency Oxygen Reduction. *Nano Lett* **2017,** *17* (3), 2003-2009.

9. Wang, M.; Yang, W.; Li, X.; Xu, Y.; Zheng, L.; Su, C.; Liu, B., Atomically Dispersed Fe–Heteroatom (N, S) Bridge Sites Anchored on Carbon Nanosheets for Promoting Oxygen Reduction Reaction. *ACS Energy Letters* **2021,** *6* (2), 379-386.
